# How Does HTLV-1 Persist in the Host?

**DOI:** 10.3390/v18101121

**Published:** 2026-10-10

**Authors:** Charles R. M. Bangham

**Affiliations:** Department of Infectious Disease, Faculty of Medicine, Imperial College London, London SW7 2AZ, UK; c.bangham@imperial.ac.uk

**Keywords:** HTLV-1, retrovirus, clonality, virological synapse, CTCF

## Abstract

HTLV-1 resists clearance from its host by the strong HTLV-1-specific immune response and by drugs. The tactics and strategies by which it does so illustrate the exquisite adaptation to the host that HTLV-1 has made in its tens of thousands of years of stable co-existence with humans. This adaptation contrasts strongly with the biology of HIV-1, which has established widespread infection in humans for only around a century. The mode of persistence of HTLV-1 makes it less pathogenic than HIV-1, despite causing serious malignant and inflammatory diseases in a proportion of people with the virus, but at the same time makes HTLV-1 harder to treat and eradicate. In this article I outline the different means that HTLV-1 has evolved to persist in the host.

## 1. Introduction

Human T-cell leukemia virus type 1 (HTLV-1), the first recognized infectious human retrovirus, was discovered in 1980 [1]. It is classified in the *Deltaretrovirus* genus, together with HTLV-2, -3 and -4 and bovine leukemia virus. The virus is widespread in areas of the tropics and subtropics [2], especially in sub-Saharan Africa, Latin America and the Caribbean, Japan, Melanesia and Australia, and in parts of the Middle East including northern Iran. The distribution is characteristically patchy: a town with a high prevalence may be close to other towns where the virus is uncommon. The total number of people in the world infected with HTLV-1 is not precisely known: it has been estimated at 5 to 10 million, but the confidence limits on this estimate are wide because systematic epidemiology is lacking in many endemic regions [2].

HTLV-1 can infect virtually any nucleated mammalian cell in vitro, but in vivo around 95% of the infected cells are CD4^+^ T cells and 5% are CD8^+^ T cells [3].

Most (~95%) people infected with HTLV-1 remain asymptomatic and unaware of the infection, but the two types of disease caused by HTLV-1 are severe and difficult to treat. Adult T cell leukemia/lymphoma (ATL) [4], a tumour typically of CD4^+^ T cells [5], develops in about 5% of people carrying the virus, usually after many years of asymptomatic carriage of the virus; the lifetime risk of ATL rises to ~20% in individuals whose proviral load exceeds 4% of peripheral blood mononuclear cells [6]. The disease is highly refractory to conventional chemotherapy, and other treatments including monoclonal anti-CD4 antibodies, AZT + type 1 interferons, and allogeneic stem-cell transplantation, have had limited success; the life expectancy of the usual aggressive form of the disease remains very low, with a median of less than one year [7].

HTLV-1 also causes a number of chronic inflammatory diseases, of which the commonest is HTLV-1-associated myelopathy [8,9,10] (HAM; myelopathy denotes spinal cord disease). HAM presents, typically in a middle-aged female, with low back pain and difficulty in walking: over months or years there is unremitting progression in muscle stiffness (spasticity) of the lower limbs, often culminating in paralysis, and the patient is often dependent on walking aids or a wheelchair after some years. Chronic inflammatory diseases have also been described in people with HTLV-1, affecting the eyes, lungs, skin, and muscles in addition to the lower limbs. These conditions differ from ATL in their frequency, pathogenesis, age, sex and population distribution, and prognosis: while the diseases themselves are not directly lethal, they are associated with a reduction in life expectancy [11].

In this article I review the currently understood tactics and strategies that HTLV-1 uses in order to persist life-long in the host. For further detail the reader is referred to recent reviews [12,13].

## 2. Mechanisms of Within-Host Persistence

### 2.1. Integration in the Host Genome

**Figure 1:**
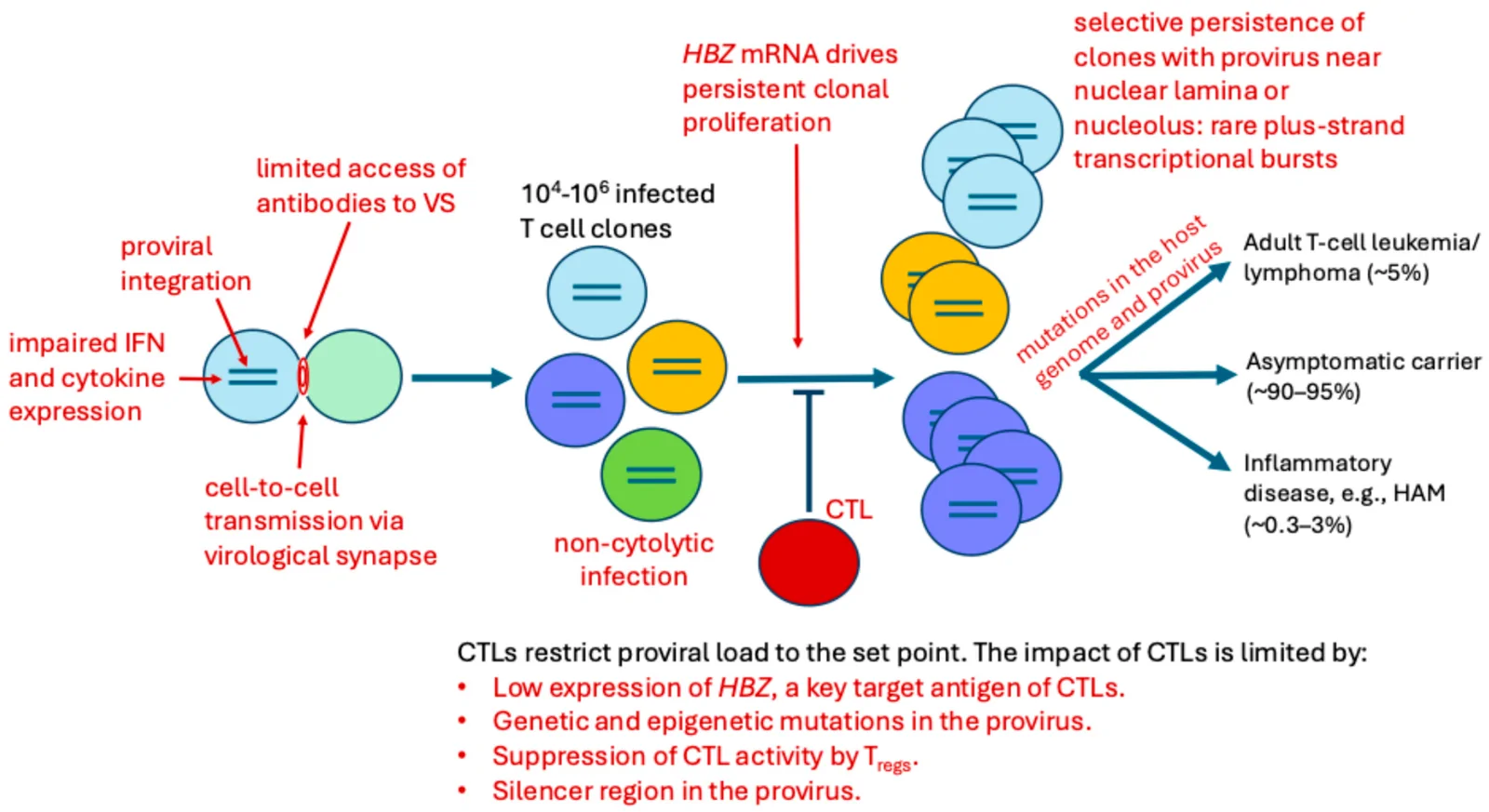
The main mechanisms by which HTLV-1 persists in the host are shown in red. The double line in a cell represents the HTLV-1 provirus in an infected cell. Each colour represents a different clone of HTLV-1-infected T cells; the dark red cell depicts a CTL.

Integration as a provirus into the host genome enables HTLV-1, like other infectious retroviruses, to persist in the cell and, while it remains untranscribed, remain undetectable by host defences (Figure 1). But the provirus must retain the capacity to be transcribed in order to transmit to a new host and to overcome the putative fitness cost of integration, which is probably small on average.

HTLV-1 integrates a single copy [14] of the provirus into the host DNA; it is not known how superinfection is prevented. The integration sites are not randomly distributed [15]: although there are no hotspots of integration, there is a weak preference for actively transcribed chromatin and for a non-palindromic DNA sequence motif of ~8 nucleotides [16]. However, the genomic integration sites in HTLV-1-infected cells that survive long-term in vivo are not randomly distributed: see Section 2.9 below.

### 2.2. Non-Cytolytic Infection

HIV-1 infection kills the host cell in the great majority of cases, although it is now clear that a small proportion of infected T cell clones can persist for long periods in the reservoir in vivo. By contrast, HTLV-1 itself is non-cytolytic, but expression of the provirus exposes the cell to destruction by the strong host immune response. The lack of cytotoxicity of HTLV-1 clearly contributes strongly to its persistence in the host.

### 2.3. Persistent Clonal Proliferation

A cardinal feature of HTLV-1 infection is a high proviral load, that is, a high proportion of peripheral blood mononuclear cells that carry a provirus; the proviral load in HIV-1 is typically at least an order of magnitude lower. In an asymptomatic HTLV-1 carrier, the proviral load is strongly correlated with the risk of developing either an inflammatory disease or ATL (or in rare cases, both), and so is of considerable prognostic significance. Soon after the discovery of HTLV-1 it became clear that a small number of infected T cell clones reach a stable and very high abundance in the blood [17,18]; each clone can be identified by the unique genomic integration site of the provirus. For many years the high proviral load was ascribed to these abundant clones, but the advent of high-throughput techniques to quantify the number—and simultaneously the abundance—of retrovirus-infected clones [15] showed that the number of distinct HTLV-1-infected T cell clones in one host is very high, ranging from ~10^4^ to over 10^6^. The proviral load, and therefore the risk of an HTLV-1-associated disease, is proportional to the number of distinct clones, not to the number of cells present in a few highly abundant clones [15]. Further, a large number of the infected T cell clones, perhaps the majority, persist for the rest of that host’s life. These long-term-surviving clones have a significant growth advantage over uninfected T cells, and so maintain the persistence of the virus in the host. This growth advantage appears to be mainly due to the actions of RNA from the *HBZ* gene, encoded on the negative strand of the provirus. The cell-proliferative actions of *HBZ* are exerted by its mRNA [19], which is largely (~90%) retained in the nucleus [20]: the restricted nuclear export maintains a very low abundance of HBZ protein, which plays a key part in minimizing the impact of the immune response (see Section 2.6 below). The *HBZ* gene products stimulate transcription of BATF3, TGF-β [21] and the cytokine IL-10 [22]: these actions contribute to the proliferation of both non-malignant HTLV-1-infected T cells and ATL cells. HBZ also enhances expression of two cell-surface proteins, CCR4 and TIGIT [23,24], which are strongly associated with the survival and function of the immunosuppressive regulatory T cells (T_regs_)—see Section 2.8 below.

### 2.4. Pattern of Transcriptional Latency and Reactivation

For most of the time, the plus-strand of the HTLV-1 provirus remains transcriptionally silent in vivo in each infected cell; it is expressed in each cell in rare, strong self-limiting bursts of transcription [20,25], probably triggered by cell stress resulting from a change in its physical or chemical environment [26]. This severely restricted expression of the plus-strand limits the exposure of the infected cell to host immune surveillance, and so makes a major contribution to persistence. By contrast, *HBZ* transcription (from the minus-strand) is detected in ~50% of infected cells in the peripheral blood at a given instant [27], but adds little to the risk of immune-mediated killing, because of adaptations in the sequence, expression and distribution of *HBZ*-encoded products—see Section 2.6 below.

The reversible transcriptional latency of the HTLV-1 proviral plus-strand is maintained by a number of factors, including epigenetic modifications of the provirus (PRC1-mediated ubiquitylation of the proviral promoter/enhancer [26]; DNA methylation [28,29]) and a silencer element in the provirus [30,31,32]; it is likely that more factors remain to be discovered.

### 2.5. CTCF

CTCF (CCCTCC-binding factor) is a zinc-finger protein which binds to a DNA motif of ~20 nucleotides that is present in tens of thousands of copies in the mammalian genome [33]. By dimerizing with a nearby copy of DNA-bound CTCF, the molecule forms a loop of chromatin, extruded through the heterotetrameric protein cohesin, and in so doing it plays a central part in structuring the genome [34] and, by regulating enhancer–promoter contacts, in controlling gene transcription [35]. Remarkably, the HTLV-1 provirus binds one molecule of CTCF [36], and so introduces an aberrant or ‘ectopic’ CTCF binding site in the unique proviral integration site in each one of the tens (or hundreds) of thousands of infected T cell clones in a single host. In this large-scale experiment of nature, these aberrant bound CTCF molecules have the potential to disrupt the structure and expression of host chromatin in a different manner in each infected clone [37].

The strong presumption must be that CTCF binding confers a significant evolutionary advantage on the virus: it strains credulity to suppose that binding of such a fundamental component of mammalian chromatin is an evolutionary accident of no benefit to the virus. However, the nature of this putative advantage is only partly understood [38]. It is probable that any advantage is conferred by local effects of CTCF within the provirus, such as its insulator function; although dimerization with CTCF in the host genome has widespread effects on chromatin structure and expression [37], any possible benefit to the virus of these consequences must be, in effect, random, because of the great diversity of proviral integration sites. However, this does not exclude the possibility that a survival or proliferation advantage can be conferred by CTCF in an occasional T cell clone through the effects of binding on host chromatin structure and transcription, although such instances may be rare.

### 2.6. Immune Evasion

The impact of innate immunity on HTLV-1 is limited by the transcriptional pattern of the virus and by specific suppressive mechanisms, so contributing to viral persistence. The cytidine deaminase APOBEC3G produces rare mutations in the plus-strand of the provirus [39], but these mutations often have little impact on the persistence of the cell, which depends largely on the minus-strand-encoded gene *HBZ*. HTLV-1 Tax actively diminishes the effectiveness of the cytokine response by at least two mechanisms: by inhibiting the kinase TBK1 [40], which (by phosphorylating IRF3) regulates production of interferon, and by inducing expression of the suppressor of cytokine signalling SOCS1 [41,42].

The cellular innate immune response to HTLV-1 is poorly understood. The frequency of circulating natural killer (NK) cells is lower in patients with HAM than in asymptomatic carriers of the virus [43,44,45], but the reasons for this observation are not known. The dendritic cell (DC) is the most potent producer of type 1 interferons, but as noted above the impact of these interferons on HTLV-1 infection and persistence is limited; the important role of the DC in the infection is to stimulate the cell-mediated response.

The most important mechanism by which HTLV-1 limits the effectiveness of host immune attack is integration of the provirus into the host genome, with the severely restricted pattern of expression of the proviral plus-strand described above. The antigens encoded on the plus-strand are highly immunogenic, especially the transcriptional transactivator protein Tax, which elicits abundant anti-Tax cytotoxic T lymphocytes (CTLs) in the peripheral blood [46,47,48]. But since at any given moment only a small proportion of infected cells express these antigens, the CTLs make a limited impact on the proviral load.

The principal function of Tax is to stimulate transcription of the proviral plus-strand in a strong positive feedback loop, to drive propagation of the virus [12]. The intermittent and short-lived HTLV-1 Tax expression in each cell enables the virus to balance counterselection by the CTL response and actions of Tax that promote the survival of the cell, and the effects of Tax on the proliferation and malignant transformation of HTLV-1-infected cells are complex and dynamic. Tax induces expression of several host genes, notably NF-κB (via activation of the cellular kinases IκB Kinase α (IKKα) and IKKβ) [49], which in turn activates transcription of anti-apoptotic genes [50]. Tax also causes a temporary, reversible arrest of the cell cycle [51,52] followed by a compensatory burst of proliferation [51]. While Tax can contribute to oncogenesis in some cases, in particular by causing genome instability through impairment of both the DNA damage response pathway [53] and DNA double-strand repair [54], ATL can arise in clones carrying a provirus that never expressed Tax, because of a mutation or deletion that occurred before proviral integration [55].

As outlined above, frequent transcription of the *HBZ* gene, from the minus-strand of the provirus, is responsible for maintaining the constant proliferation of HTLV-1-infected T cell clones in vivo, which is the chief mechanism by which the virus persists in the individual host. Because of this central function of HBZ in maintaining viral persistence, the immune response to this protein—again, in particular the CTL response—is of special importance [56]. The virus has therefore (teleologically speaking) evolved to restrict the expression of *HBZ* to the minimal level required. Each infected cell, which expresses *HBZ* about 50% of the time, contains typically between two and six copies of *HBZ* mRNA [20]; the mRNA itself plays a part in regulating cellular proliferation, and its export into the cytoplasm is also highly restricted, thereby limiting the capacity for translation of HBZ protein. Finally, the CTL epitopes (antigenic determinants) on the HBZ protein are weakly immunogenic, in strong contrast with those on the Tax protein.

As a consequence of these adaptations of *HBZ*, the frequency of HBZ-specific CTLs in the blood is very low; however, both genetic and functional evidence shows that these CTLs play a significant part in limiting the steady-state proviral load and in reducing the risk of the inflammatory disease HAM.

The importance of the CTL response in regulating HTLV-1 replication is shown by three observations. First, the CTLs found in peripheral blood are not resting memory cells, but are chronically activated and can kill HTLV-1-expressing cells immediately [48]. The chronic activation demonstrates that the cells have recently been exposed to newly produced antigen in vivo. Second, impairment of the CTL response quickly results in an increase in the proviral load. Third, specific HLA class 1 alleles, which determine the specificity and efficacy of CTLs, are associated with a lower proviral load and a lower risk of developing the inflammatory disease HAM [57]. Thus, as in other persistent viral infections such as EBV [58] and other herpesviruses, an equilibrium is established between the constant attempt of the virus to replicate and the chronically activated, virus-specific T cell response.

### 2.7. Cell-to-Cell Propagation: The Virological Synapse

Infectious, cell-free virions of HTLV-1 are virtually undetectable in the peripheral blood and in the supernatant above HTLV-1-infected T cells cultured in vitro; the few particles released in in vitro cultures are poorly infectious. It is now understood that HTLV-1 does not propagate by releasing cell-free particles, but depends on transmission by direct contact between an infected T cell and a new host cell: infection of a new host requires transfer of infected T cells (through breastfeeding, sexual intercourse, transfusion or transplantation), and transfusion of cell-free blood products does not transfer the infection. Dendritic cells have been shown to be infected by cell-free HTLV-1 in vitro [59], but since the cell-free virus is rare or undetectable, the importance of this in vivo is in doubt. Driven by the HTLV-1 Tax protein and strongly promoted by binding of adhesion molecule ICAM-1 on the infected cell and its partner LFA-1 on the target cell, the cell-to-cell contact results in formation of a structured interface called the virological synapse (VS) [60].

The VS contains organized concentric domains of adhesion molecules, and the structure strongly resembles the immunological synapse—the cell-to-cell contact that mediates T cell functions. But in HTLV-1 infection, this process is driven not by antigen recognition, but by the virus itself. The cell contact triggers polarization of the infected T cell’s microtubule cytoskeleton, bringing the microtubule organizing centre (MTOC) to lie adjacent to the synapse; the microtubules transport viral proteins to the synapse, where enveloped virions are assembled, bud from the plasma membrane and cross a small gulf between the two cells to engage the cellular receptors on the target cell. Some virions may be transferred at the periphery of the synapse [61], but many cross a pocket that is enclosed by the close apposition of the plasma membranes: these particles are therefore isolated from the extracellular fluid and cannot be reached by antibodies. Thus, HTLV-1 has evolved to substitute the mobility of the host cell for the mobility of cell-free particles, and by transmitting directly from cell to cell it limits the impact of the antibody response.

### 2.8. Regulatory T Cells (T_regs_)

T_regs_ are defined by their ability to suppress the activity of other T cells. This group of CD4^+^ T cells consists of different subsets, each with a characteristic set of surface molecules and immune-suppressive mechanisms. The largest population expresses FoxP3 and the high-affinity IL-2 receptor CD25 on the cell surface, together with other characteristic markers such as TIGIT. A high frequency of these classical T_regs_ is found in the peripheral blood in HTLV-1 infection [62], probably as an adaptation to reduce the effectiveness of the host T cell response to the virus and so aid its persistence. The virus elicits and maintains this high frequency of T_regs_ by at least two mechanisms. First, HBZ drives production of TGF-β, which in turn increases the expression of the T_reg_-associated protein FoxP3 [63]. Second, the Tax protein drives production of the chemokine CCL22, which binds the surface receptor CCR4 expressed on the T_regs_, maintaining the survival of the immunosuppressive cells in the blood [64].

As a consequence of these diverse mechanisms by which HTLV-1 limits effective immune surveillance, there is less selection pressure to mutate T cell epitopes to evade immune-mediated killing. Mutations that lead to failure of autologous CTL recognition have been observed [65], but this mechanism is of minor importance compared with that in HIV-1 infection. The rate of evolution of HTLV-1 is correspondingly low [66], and the nucleotide sequence and antigenic diversity of the different strains are much smaller than those of HIV-1.

### 2.9. Selective Clonal Persistence of HTLV-1-Infected Cells

Although the integration sites of the HTLV-1 provirus in the host genome are not randomly distributed, the relatively weak preferences for open chromatin and a primary DNA motif result in a very wide distribution of sites. In cells infected with HTLV-1 in vitro (by contact with an HTLV-1-expressing cell), the average frequency of integration in each chromosome, over a large number of clones, is simply proportional to the size of that chromosome. By contrast, the chromosomal distribution of proviruses in the infected T cell clones found in vivo is strongly biased [67], with particularly frequent events in the acrocentric chromosomes. This observation suggested that the characteristics of these integration sites would reveal more tactics by which HTLV-1 persists in the host.

By comparing the integration sites of large numbers of clones infected in vitro with those observed in vivo, we found a very strong correlation between the probability of clonal survival in vivo and the spatial position of the provirus in the nucleus [67]. That is, a T cell clone that carries the provirus in chromatin that normally lies near the nuclear lamina or near the periphery of the nucleolus is much more likely to survive in vivo than one in which the provirus lies in the centre of the nucleus. Independently, clones with a provirus near a telomere are strongly counterselected in vivo.

These correlations accounted for a remarkable 41% of the selective survival of HTLV-1^+^ clones in vivo. The same factors—the position of the provirus in the nucleus and within a chromosome—were also statistically significant in HIV-1-infected cells isolated from peripheral blood, but only accounted for 7% of their selective survival [67].

These nuclear locations are associated with heterochromatin and transcriptional silencing. However, the observed correlation was independent of the average transcription intensity of the host chromatin immediately flanking the provirus, which itself was correlated with survival: actively transcribed chromatin nearby leads to counterselection of that clone. To reconcile these observations, we hypothesize that there is a correlation between the frequency of gene bursting and the radial intranuclear position of the gene in normal mammalian cells. The idea is that a cell that expresses rare and short-lived—albeit intense—bursts of the HTLV-1 plus-strand, from a provirus near the nuclear lamina, is less exposed to immune counterselection, over time, than a cell whose provirus in the centre of the nucleus is frequently expressed. To test this hypothesis, data are needed on the frequency of transcriptional bursts of many genes in a large number of single cells.

## 3. Robin Weiss and HTLV-1

Robin Weiss laid the groundwork for much subsequent research on HTLV-1, especially on the cellular tropism of the virus and the anti-HTLV-1 antibody response. His experience and expertise on endogenous and exogenous viruses and cancer (see below) placed him in a strong position to work on HTLV-1, following the publication of its discovery by Gallo and colleagues in 1980 [1].

Weiss’s work on retroviruses began before the discovery of reverse transcriptase and extended to the emergence of infectious human retroviruses and the development of effective therapy for HIV-1. In his early work on Rous sarcoma virus (RSV) he made the startling observation [68] that a defective strain (Bryan) of RSV could be rescued by a factor apparently present in the normal cell. His postulate, that this factor was a retroviral (Env) element in the human genome, was dismissed as ‘impossible’ by one reviewer. But Temin was working and thinking along similar lines and, when visiting Weiss in London in the late 1960s—before his publication of the discovery of RT—he encouraged Weiss to persevere in attempts to publish his results.

These observations, and his subsequent work on avian leukosis virus, established Weiss’s long interests in endogenous [69] and exogenous retroviruses and in cancer. He developed unusual expertise in designing and making new experimental techniques and serological assays for retroviruses. A particularly productive technique was the development of pseudotype viruses, in which the receptor-binding G protein of the vesicular stomatitis virus was replaced with the Env protein of a retrovirus.

Weiss’s group developed serological and receptor-binding assays, often using pseudotype viruses [70,71,72]: antibody-mediated neutralization of the VSV/HTLV-1 Env pseudotype was sustained at high titres throughout the infection, but was unrelated to disease risk. He then studied sequence variation in HTLV-1 [73] and the properties of the HTLV-1 transcriptional transactivator protein Tax [74], and had a productive collaboration with the hematologist Catovsky and his colleagues [75,76] in research on the HTLV-1 malignancy, adult T cell leukemia/lymphoma. This work in turn enabled Weiss to make critical contributions from the outset to the study of HIV, which was quickly emerging at the same time and was formally identified in 1983.

Weiss’s training in zoology and his long trajectory in retrovirology and cancer research gave him a uniquely broad and deep perspective on their evolution, biology and pathology, and this perspective shone through in his writing and his seminars.

## Data Availability

Data availability is indicated in the respective publication cited; no new data are presented in this article.
